# Climate stressor projections inform adaptation needs in South Asian oilseed systems

**DOI:** 10.1038/s44264-026-00170-9

**Published:** 2026-06-30

**Authors:** Anasuya Barik, Paresh B. Shirsath, Roshan Babu Ojha, Vinay Kumar Sehgal, Virender Singh Bhatia, Sanjoy K. Bandyopadhyay, Pramod K. Aggarwal

**Affiliations:** 1 Borlaug Institute for South Asia (BISA), International Maize and Wheat Improvement Centre (CIMMYT), New Delhi, India; 2 National Agricultural Environment Research Centre, Nepal Agricultural Research Council, Lalitpur, Nepal; 3 Division of Agricultural Physics, ICAR-Indian Agricultural Research Institute, New Delhi, India; 4 Division of Plant Physiology, Indian Institute of Soybean Research, Indore, Madhya Pradesh India

**Keywords:** Climate sciences, Plant sciences

## Abstract

Climate stressors impact major oilseed crop systems of groundnut, mustard, and soybean in South Asia. Our analysis shows that the intensity of all heat-related and water stresses is projected to rise by the 2050 s and 2080 s, while rainfall-related stressors show mixed and uncertain responses. We also find that heat stress effects during the full crop cycle and the reproductive phases are different in nature. Critical-phase heat stress is likely to increase mainly in frequency rather than intensity, whereas full-cycle heat stress is likely to intensify in the future. These shifts in climate stressors have direct implications for the suitability of adaptation interventions for oilseed systems. Genetic options, such as stress-tolerant varieties, and financial instruments, such as crop insurance, emerge as consistently robust across scenarios. In contrast, structural, nutrient, and irrigation-based interventions lose effectiveness as climate stressors exceed their adaptive limits. By mapping these future suitability transitions, this study provides a first-order basis for tailored adaptation planning and climate-smart oilseed systems in South Asia.

## Introduction

Oilseeds are important from both a food and economic perspective in South Asia. Mustard, groundnut, and soybean together account for about 85% of the region’s oilseed area^1,2^. Climate stressors such as extreme heat, prolonged dry spells, erratic rainfall, and moisture deficits are expected to intensify under future warming, affecting the phenology and yield stability in these crops^3,4^. In particular, heat stress during critical reproductive phases can impair pollen viability and fertilization, and water stress during vegetative growth limits biomass accumulation^5–7^. Extreme rainfall can exacerbate soil waterlogging or nutrient loss in oilseed systems^8,9^.

Studying these stress dynamics in oilseeds is particularly crucial in South Asia. The region’s tropical climate, strong monsoon dependence, and high reliance on rainfed systems increase exposure to climate variability^10–12^. Heatwaves have become longer and more intense across the Indo-Gangetic Plains, and monsoon rainfall variability has increased in both spatial and temporal extremes^13^. The oilseed sector is also important from an economic perspective. India, for example, is one of the largest edible oil importers globally, importing over 16.5 Mt in 2022–23 to meet 55–60% of demand^14^. Enhancing domestic production by reducing climate-driven yield losses is therefore central not only to agricultural sustainability but also to economic policy and food security.

However, despite extensive research on climate impacts on oilseed crops like mustard, groundnut and soybean, most assessments remain limited in their ability to capture physiologically meaningful stress on a large spatial scale. Large-scale climate studies, such as^15^ and^16^, typically rely on coarse seasonal averages or generic thermal thresholds^4^. These thresholds do not reflect crop-specific sensitivities, while controlled-environment experiments, which derive most physiological thresholds, are conducted on small samples under idealized conditions^6,17,18^. Such laboratory-based studies are insufficient for understanding variability across larger regions, such as South Asia. Another gap in existing studies is the emphasis on stressor intensity. Most analyses neglect frequent, moderately intense events, which, particularly during reproductive phases, can be just as damaging as rare extreme peaks^6,19^.

Recent literature has evaluated the impacts of technological interventions for South Asian agriculture systems^20–23^. However, studies on oilseeds remain limited and fragmented. Agronomic interventions such as mulching, broad-bed and furrow systems, conservation tillage, and precision nutrient management are mostly tested in isolated field trials without linking their performance to projected heat, drought, or rainfall extremes^21,24^. Genetic improvements, including the development of heat- and drought-tolerant varieties, are advancing^25^, yet few studies assess where these efforts need to be targeted, given future warming scenarios. Nutrient management measures are similarly evaluated in various field studies^26,27^ without integrating future climate scenarios. Financial risk-management tools, particularly crop insurance, remain largely absent from oilseed-focused climate analyses despite their relevance for buffering increasing production volatility^28^.

To address these gaps, this study integrated physiology-informed thresholds with large-scale, multi-model climate analyses, resulting in accurate estimates of the intensities and frequencies of stressors in mustard, groundnut, and soybean systems in South Asia. Then, we developed a framework to link climate stressors and assess the effectiveness of adaptation interventions in the region. The specific objectives of the study were: (1) to quantify and project the intensity–frequency patterns of key climate stressors observed and projected climate data, (2) to differentiate between full-cycle (cardinal) and critical-phase (pollination) stresses, and (3) to evaluate the current and future suitability of genetic, agronomic, structural, and financial interventions to guide targeted adaptation planning.

## Results

### Projected shifts in the intensity of climate stressors

**Fig. 1:**
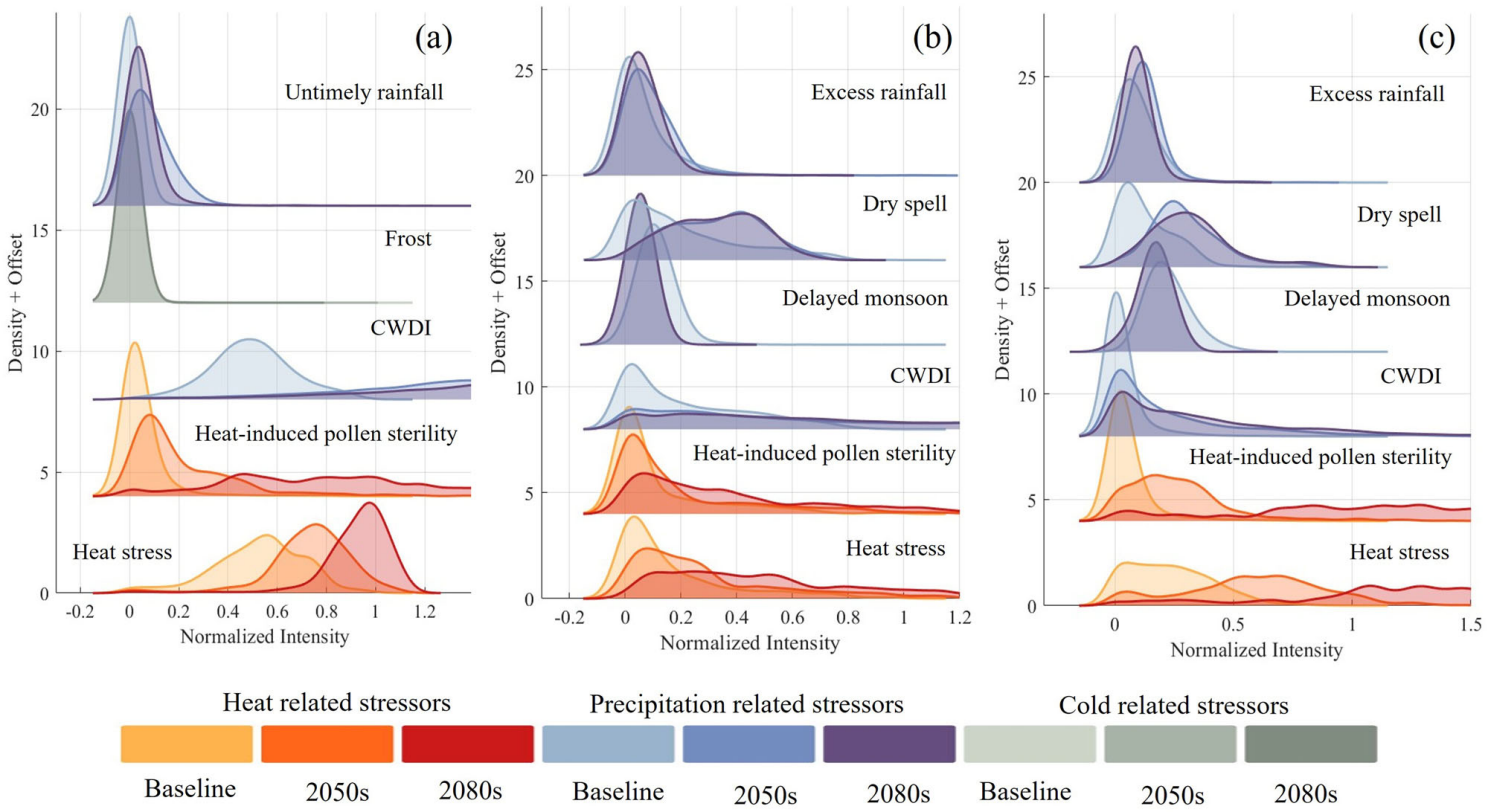
Kernel density distributions of climate stressor intensities for major oilseed crops under baseline and future climate scenarios. Ridge plots show kernel density estimates (KDE) for intensity distributions of climate stressors in (**a**) mustard, (**b**) groundnut, and (**c**) soybean in baseline and under the high-emissions SSP5-8.5 scenario. The y-axis defines the kernel densities with an offset of stressor order x 4 units. CWDI refers to Crop Water Deficit Index.

The distribution of the climate stressor intensities in baseline and projections under the SSP5-8.5 high-emissions scenario (2050 s, 2080 s) for mustard, groundnut, and soybean is summarized in Fig. 1. Heat-related stressors (shown in orange-red) shift to the right, indicating increasing intensity in future periods. Among these, mustard exhibits the highest normalized intensity for heat stress, which is projected to increase further, reflecting increasing thermal exposure during its winter crop cycle. The cold-related stressor of frost (green panel), relevant only for mustard, shows negligible intensity at baseline and remains low in the future high-emission scenario.

Precipitation-related stressors (shown in blue-purple) also show significant changes. For groundnut and soybean (Fig. 1b, c), dry spell intensities shift rightward, especially in the 2080 s, suggesting more intense rainfall variability under future climate conditions. The intensity peaks for delayed monsoon, however, shift leftward in future periods, indicating a reduced likelihood of delayed-onset events. This suggests that the monsoon onset may occur earlier or closer to the baseline mean onset in the future climate. Peaks for untimely or excessive rainfall show a slight increase in the future curves. Across all three crops, the CWDI curves shift to the right. This indicates an increasing crop water deficit in future periods despite projected increases in precipitation. This can be attributed to the combined influence of higher evapotranspiration demand and imbalanced soil–water dynamics under warmer conditions.

### Temporal evolution and ranking of climate stressor exposure across crops

**Fig. 2:**
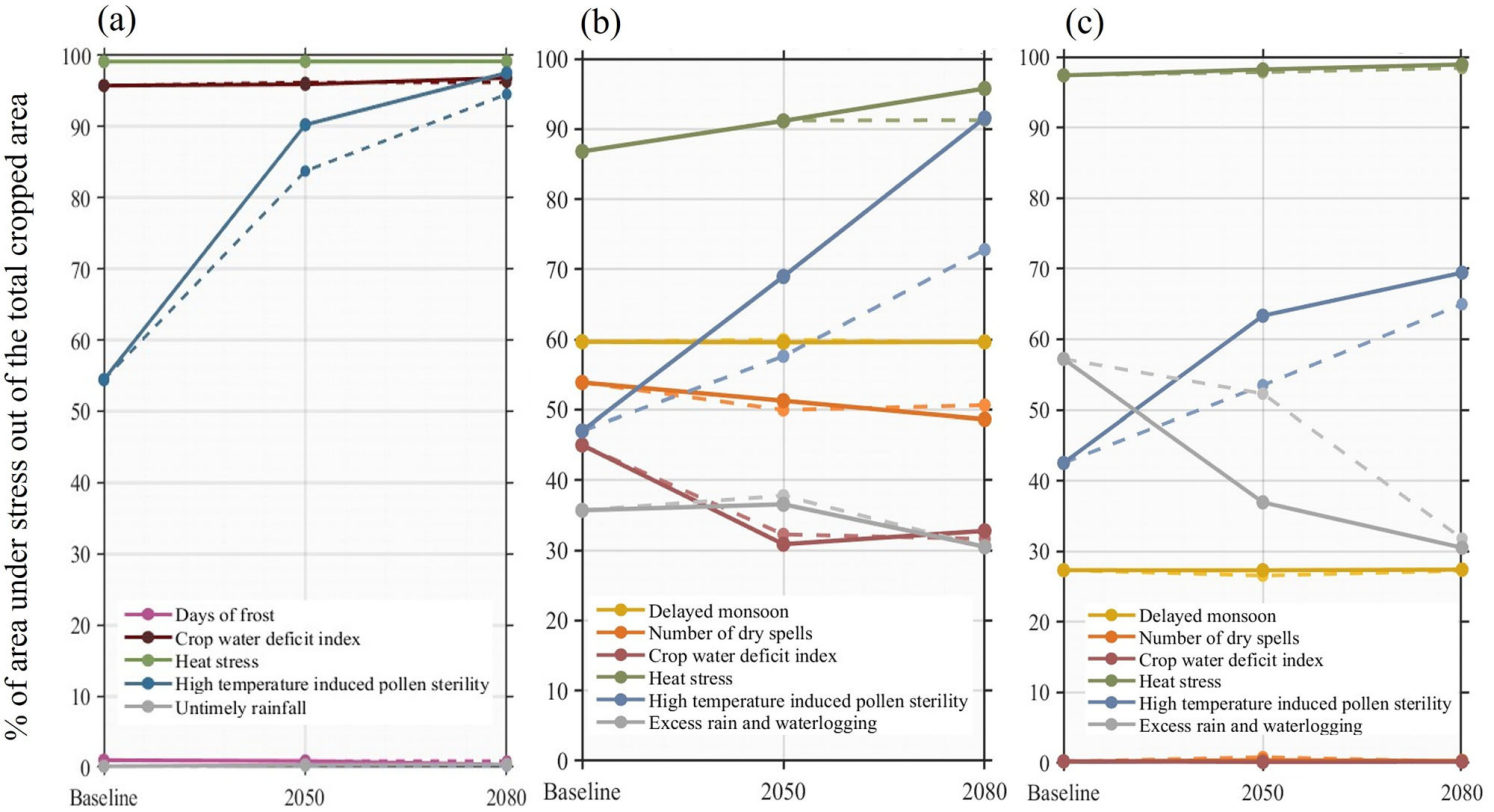
Temporal shifts in cropped area exposure to climate stressors under moderate and high emissions scenarios in future. Ranked bump charts showing temporal changes in the percentage of cropped area exposed to climate stressors from baseline to 2050 s and 2080 s, under SSP2-4.5 (dashed lines) and SSP5-8.5 (solid lines) scenarios for (**a**) mustard, (**b**) groundnut, and (**c**) soybean.

The ranking bump charts show the dominant stressors spatially at each period, and their relative importance shifts under climate change scenarios (Fig. 2).

In mustard (Fig. 2a), heat stress and the crop water deficit index (CWDI) stressors affect the largest percentage of mustard-growing regions in the baseline and remain consistently high in both the 2050 s and 2080 s under SSP2-4.5 and SSP5-8.5 scenarios. The persistently high CWDI reflects low precipitation during the crop’s rabi-season growth period. However, mustard is largely irrigated, so this water deficit does not directly translate into yield stress. These two stressors are followed by high-temperature–induced pollen sterility, which affects about 54% of mustard-growing areas. In future scenarios, the area under this stressor increases sharply by about 60% and 80% in the 2050 s and 2080 s, respectively, in both scenarios, becoming nearly comparable to CWDI and heat stress. Frost and untimely rainfall affect only a small proportion of the cropped area in the baseline period and are projected to decline further under both future scenarios.

In groundnut (Fig. 2b), heat stress affects the maximum crop-growing area in the baseline. Its dominance intensifies in the 2050 s and 2080 s, with a steeper rise under SSP5-8.5 than under SSP2-4.5. This is followed by delayed monsoon, dry spells, and crop water deficit index (CWDI), with each affecting about 30–60% of the groundnut-growing regions in the baseline. CWDI shows a decline by the 2050 s and a slight recovery by the 2080 s, but overall decreases across both scenarios. High temperature–induced pollen sterility, though lower in the baseline, is expected to affect more than 90% of the cropped area by the 2080 s under SSP5-8.5. Excess rain and waterlogging show a modest rise in the 2050 s, followed by a decline thereafter. Most stressors exhibit unidirectional, scenario-consistent increases between SSP2-4.5 and SSP5-8.5, with pollen sterility showing the steepest rise under high emissions. In contrast, delayed monsoon projections diverge across scenarios, showing opposite directional changes by the 2080 s, suggesting lower confidence in future projections. Overall, groundnut systems are expected to experience increasing heat-related stressors, with high confidence in the intensification of thermal risks and lower confidence in rainfall-related stressors.

In soybean (Fig. 2c), cardinal heat stress affects over 95% of the cropped area in the baseline and is projected to further intensify by the 2050 s and 2080 s. Both SSP2-4.5 and SSP5-8.5 scenarios show agreement on this rising trajectory. Excess rainfall and waterlogging affect more than 50% of soybean areas. However, this stressor is projected to decline sharply under SSP5-8.5 and more gradually under SSP2-4.5, stabilizing at around 30% of the affected area by the 2080 s in both scenarios. The delayed monsoon ranks next, affecting roughly 30% of the cropped area in the baseline. Similar to groundnut, the area under this stressor is expected to undergo minimal change in the future. In contrast, high-temperature–induced pollen sterility, which initially affects about 40% of soybean areas, is expected to increase sharply in the future, particularly under SSP5-8.5. Meanwhile, dry spells and CWDI affect minimal area shares at baseline and remain negligible across future periods.

A consistent observation across all three oilseed crops is that high-temperature–induced pollen sterility is projected to affect more area under future climates. To further understand the distinct behavior of critical-phase heat stress, we compared full-cycle (cardinal) and pollination-phase heat stress using bivariate intensity–frequency maps.

### Bivariate intensity-frequency analysis of heat-related stress

**Fig. 3:**
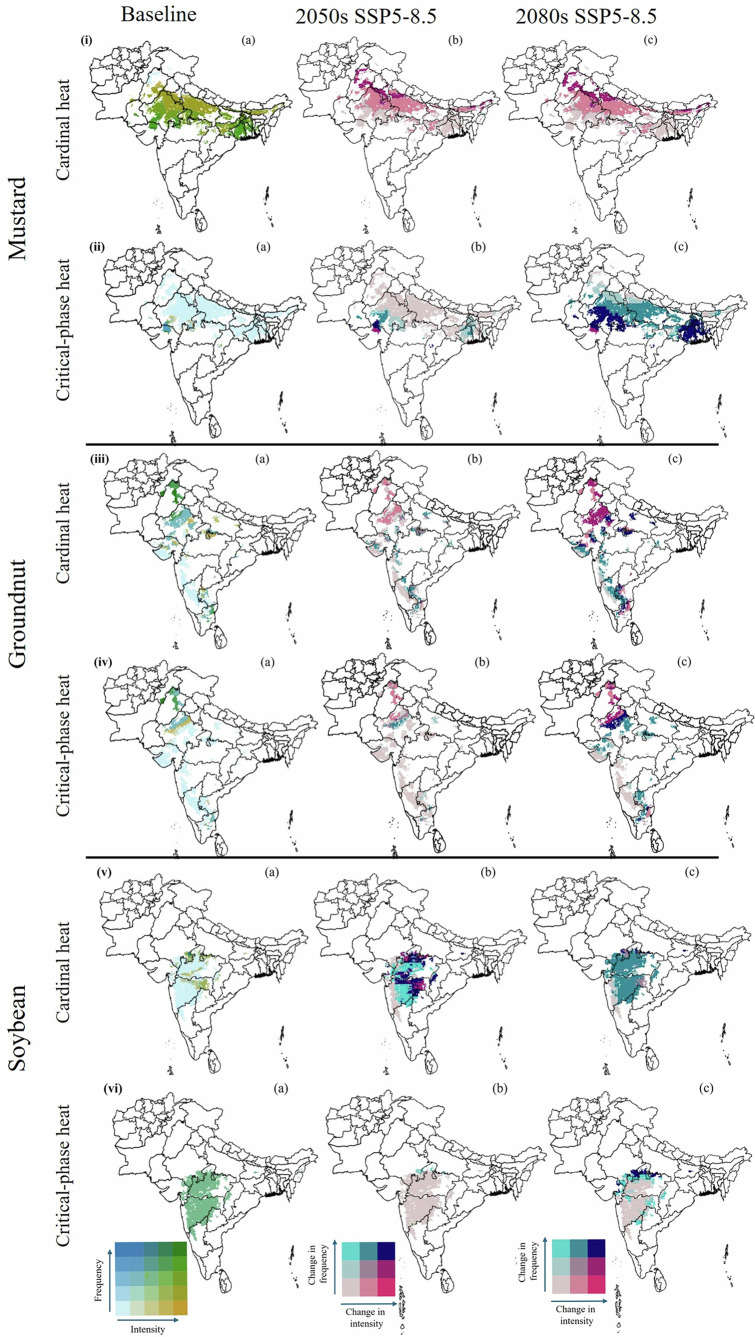
Spatial patterns of heat stress intensity and frequency across oilseed crops under baseline and future climates. Bivariate choropleth maps showing spatial distribution of heat stress intensity and frequency for mustard (i, ii), groundnut (iii, iv), and soybean (v, vi) under (**a**) baseline conditions and projected changes in (**b**) 2050 s and (**c**) 2080 s for SSP5-8.5 scenario. For each crop, the first row represents full-cycle (cardinal) heat stress, and the second row represents critical-phase heat stress during the pollination phase of crop growth.

In mustard (Fig. 3a(i)), the baseline full-cycle heat stress shows high intensity and frequency, concentrated in the western and northern growing regions. Critical-phase heat stress during pollination (Fig. 3a(ii)), however, is characterized by a higher frequency relative to intensity in 9% of the crop growing area, with low intensity-frequency spread more heterogeneously across the mustard belt. Future projections indicate a sharp increase of full-cycle heat stress intensity in 98% of the area, particularly in northern regions (Fig. 3b(i), 3c(i)). In comparison, critical-phase heat stress (Fig. 3b(ii), 3c(ii)) shows increases in frequency than in intensity in 90% of the area, especially at the western and eastern parts of the mustard-growing regions by the 2080 s. This divergence demonstrates that cumulative seasonal heat exposure and acute reproductive-stage stressors evolve differently under warming.

Groundnut shows a similar pattern in full-cycle heat stress and critical phase stressors in the baseline (Fig. 3a(iii), 3a(iv)). We observe higher intensity of both the stressors in northern Pakistan and the Rajasthan state of India, and higher frequency in the groundnut-growing regions of the southern peninsular in India. In future projections, the patterns of change in cardinal- and critical-phase heat stress differ significantly. The full-cycle heat stress intensity increases further in the north, whereas southern regions with already frequent events experience additional increases in frequency (Fig. 3c(iii)). However, projected changes in critical-phase heat stress are dominated by increases in frequency across Southern India (Figs. 3b, 2c(iv)), indicating that reproductive-stage heat risk may escalate through repeated moderate events rather than extreme peaks.

Soybean exhibits moderate full-cycle heat stress intensity at baseline in most of the crop-growing regions (Fig. 3a(v)), with about 26% of the crop area governed more by high-intensity events than by high-frequency occurrences. Future projections highlight that frequency increases dominate, whereas intensity increases are observed in only a few localized pockets in northern and central India (Fig. 3b, 3c(v)). Critical-phase heat stress during pollination shows higher intensity-frequency than cardinal heat stress in the baseline in about 37% of the cropped area (Fig. 3a(vi)). However, cardinal heat stress is projected to increase in both intensity and frequency in the future. Critical-phase heat stress shows minimal change in the 2050 s (Fig. 3b(vi)) but is projected to increase in frequency by nearly twofold by the 2080 s (Fig. 3c(vi)). Overall, across all three crops, we observe that the changes expected in response to heat stressors during the critical reproductive stage are largely frequency-driven.

### Spatial distribution and future suitability of selected adaptation interventions

**Fig. 4:**
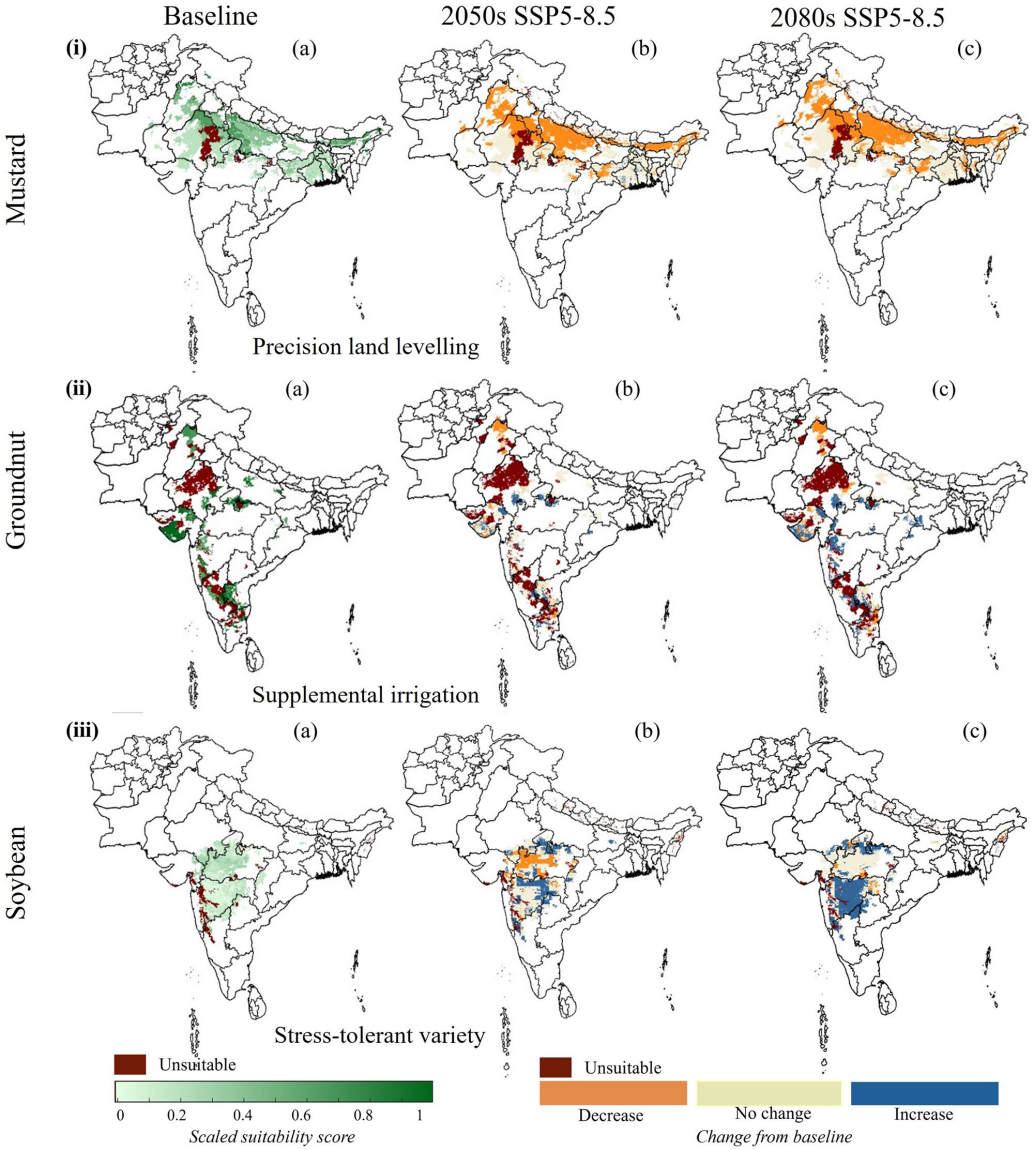
Projected changes in suitability of key adaptation interventions under future climate scenarios. Spatial distribution of (**a**) baseline suitability and projected changes in the (**b**) 2050 s and (**c**) 2080 s for selected adaptation interventions: (i) Precision land levelling (PLL) for mustard, (ii) Supplemental irrigation (SUPIR) for groundnut, and (iii) Stress-tolerant variety (STV) for soybean under SSP5-8.5.

Baseline suitability and projected changes for three widely practiced adaptation interventions (Precision Land Levelling (PLL) for mustard, Supplemental Irrigation (SUPIR) for groundnut, and Stress Tolerant Variety (STV) for soybean, mustard, groundnut, and soybean) under the SSP5-8.5 scenario were selected as they are widely adopted in the region (Fig. 4). These options were also chosen to study how different categories of interventions may respond to future climate conditions. PLL addresses soil and water management, SUPIR enhances water availability, particularly in rainfed systems, and STV provides genetic resilience against climate stress.

Precision Land Levelling (PLL) involves laser-assisted leveling to create uniform fields, improving water distribution, irrigation efficiency, and fertilizer use. In the baseline, high suitability of PLL is observed in mustard-growing regions of the Indo-Gangetic Plain (Fig. 4i(a)). This also aligns with the understanding that PLL is economically and operationally feasible mainly in the plains. This intervention is not suitable or cost-effective in the complex topography region where slope is a limitation. PLL works best under low to moderate water stress. It improves soil moisture and helps crops use water efficiently. However, when stress levels are very high, PLL alone cannot fully offset the damage, and its effectiveness declines. By the 2050 s and 2080 s, PLL suitability decreases across many areas as stressors intensify.

Supplemental irrigation (SUPIR) supports groundnut production by providing small, timely water inputs that buffer short-term moisture deficits in rainfed systems. This intervention is unsuitable in areas with very light soil, in temperate zones, and in mid- to high-hill topography due to physical constraints and is not relevant in irrigated systems. In flood, waterlogging, and high-temperature-induced pollen sterility situations, SUPIR is ineffective because such hazards cannot be mitigated through additional irrigation. In the baseline, high suitability for SUPIR is found in areas where all major stressors, such as CWDI, heat stress, delayed monsoon, and dry spell, are at manageable levels. For delayed monsoon and dry spells, SUPIR suitability peaks in moderate stress, but drops at both extremes, reflecting that it is less needed under minimal stress and largely ineffective as stress becomes very high. However, in the case of CWDI and heat stress, SUPIR maintains medium suitability even at very low and very high as additional moisture helps buffer both moderate and some extreme stress conditions. In the future, SUPIR suitability is expected to increase in pockets where CWDI and heat stress shift from low to moderate levels. This creates a new demand for seasonal watering. In contrast, suitability decreases in regions where heat stress and drought hazards escalate into very high categories. Thus, SUPIR remains most effective as an adaptation for moderate climate stress, but its utility decreases as climatic stressors intensify into very high categories.

Stress-tolerant varieties (STVs) enhance resilience to heat, drought, and other climate stresses by improving physiological and genetic traits that perform better under stress. Similar to SUPIR, STVs show maximum suitability at moderate stress levels but low suitability at very low and very high stress levels. In the baseline, STV suitability is moderate across most soybean-growing regions, reflecting a balance of manageable stress levels. Unsuitable areas occur along coastal zones and in mid- to high-elevation regions, where temperature and humidity conditions fall outside optimal thresholds. By the 2050 s, suitability for STV increases in some southern pockets, while regions in central India show a decline due to rising stress beyond the optimal range. By the 2080 s, suitability increases again across large parts of the soybean belt, as many regions transition from low- to moderate-stress categories, where STVs are most effective.

Together, the suitability patterns of PLL, SUPIR, and STV reveal key insights into adapting oilseed systems across South Asia. A major learning is that the effectiveness of all these interventions declines as stressors intensify beyond moderate levels. In the future, PLL becomes less effective as stressors intensify. SUPIR loses effectiveness as drought and heat hazards become very high, but remains effective in moderate-stress areas. STV (genetic intervention) will become increasingly effective under moderate stress conditions in soybean systems in the future. Together, these patterns emphasize that adaptation strategies must be tailored to local stress profiles and that no single intervention can mitigate future climate stressors universally.

### Changes in the area under adaptation suitability

**Fig. 5:**
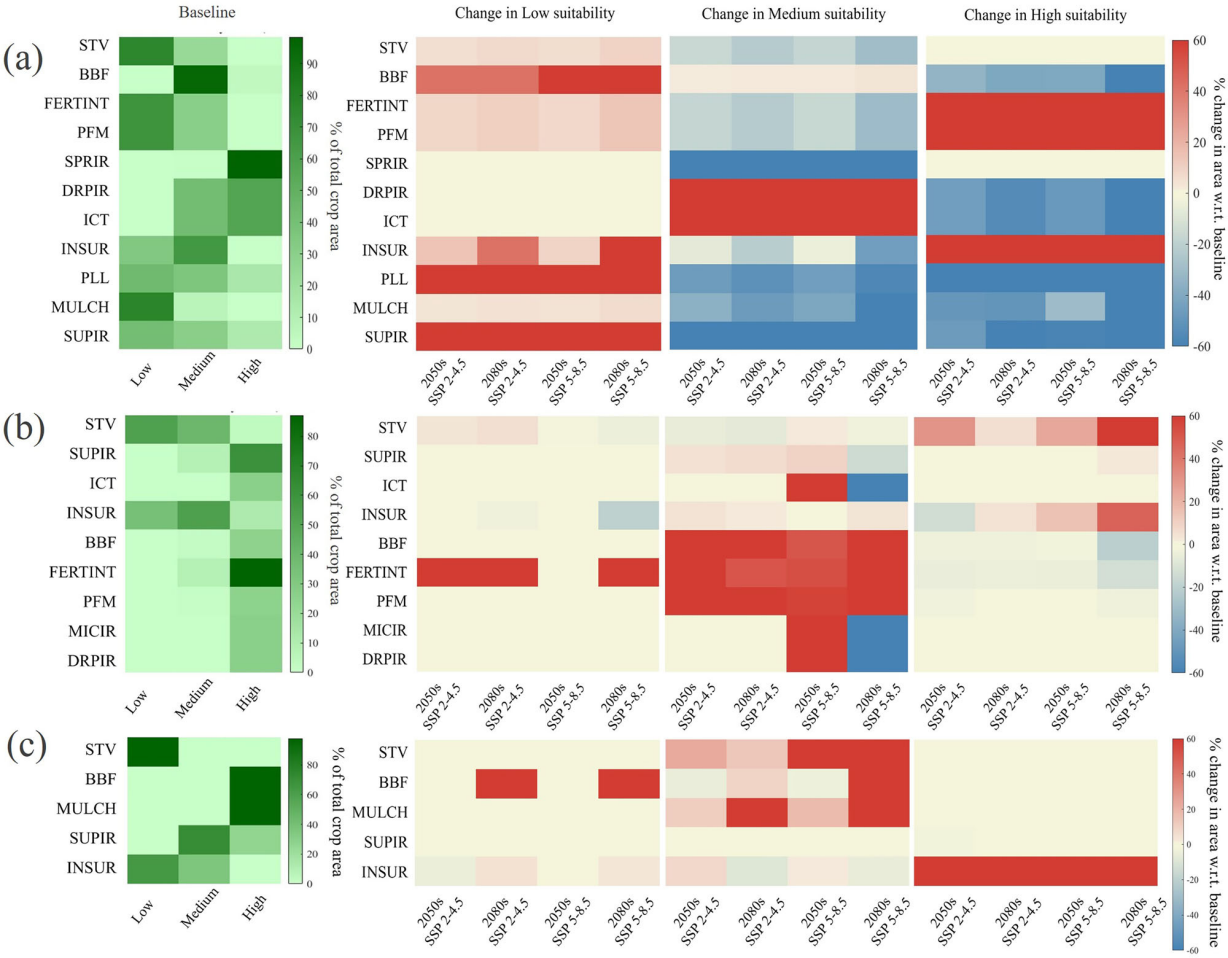
Changes in suitability distribution of adaptation interventions under future climate scenarios. Heatmaps showing baseline distribution of cropped area under low, medium, and high suitability for adaptation interventions and percentage changes in suitability area under SSP2-4.5 and SSP5-8.5 for (**a**) mustard, (**b**) groundnut, and (**c**) soybean. The adaptation interventions included in the analysis are Stress-tolerant varieties (STV), Broad-bed and furrow (BBF), Fertilizer intensification (FERTINT), Supplemental Irrigation (SUPIR), Crop Insurance (INSUR), Precision Fertilizer Management (PFM), Sprinkler Irrigation (SPRIR), ICT linked management (ICT), Precision Water Management (DRPIR), Precision land levelling (PLL), and Mulching (MULCH).

The baseline distribution of crops such as mustard, groundnut, and soybean under low, medium, and high suitability criteria for each intervention across future scenarios is presented in Fig. 5a, left-hand column. For mustard, interventions such as sprinkler and precision water management are estimated to be highly suitable in more than 60% of the cropped area. Broad-bed and furrow and stress-tolerant varieties dominate in the medium- and low-suitability classes. For groundnut, fertilizer intensification shows the highest baseline suitability, with more than 90% of the cropped area in the high suitability category. Supplemental irrigation, crop insurance, and stress-tolerant varieties also have strong baseline relevance in groundnut cropping systems. In soybean, baseline suitability is highest for agronomic and structural interventions such as broad-bed and furrow, and mulching. These interventions show more than 80% of the area in the high category of land-climate suitability. stress-tolerant varieties also shows a prominent area in the low suitability class.

Columns 2–4 in Fig. 5 present the percentage change in the area under low, medium, and high suitability classes for the adaptation interventions between the baseline and four future scenarios (SSP2-4.5–2050 s, SSP2-4.5–2080 s, SSP5-8.5–2050 s, SSP5-8.5–2080 s). Two key aspects are analyzed: (i) the direction of change in suitability classes, which indicates whether an intervention is projected to become more or less effective in future climates; and (ii) the degree of scenario alignment, which reflects the extent to which suitability changes remain consistent across emission pathways.

For mustard, fertilizer-based interventions like fertilizer intensification and precision fertilizer management show a clear increase in area under high suitability, with a decline in the medium class (Fig. 7b). Quantitatively, high suitability areas increase by about 60% relative to baseline across all four scenarios. This suggests that these fertilizer management techniques are likely to remain broadly effective under future climates. The consistency across scenarios indicates low scenario dependence. Financial interventions such as crop insurance also show strong positive shifts. The high-suitability area for insurance increases by more than 60% across all scenarios, while the medium- and low-suitability areas decline. Among structural interventions, both broad-bed and furrow and mulching show substantial increases in the low-suitability class (~60%), with a reduction in the high-suitability fraction. Though this observation is consistent in precision land levelling for both scenarios, broad-bed and furrow shows a difference in the degree of change. Overall, the suitability trajectories show that interventions linked to nutrient management and financial protection are projected to remain robust across emission pathways. At the same time, structural and some irrigation-based options become increasingly constrained under future climate stressors. Scenario alignment analysis further reveals that fertilizer-related and financial interventions exhibit low scenario dependence—that is, their relative advantage persists across emission trajectories. In contrast, irrigation and structural techniques exhibit high scenario dependence, with contrasting outcomes under SSP2 and SSP5.

**Fig. 6:**
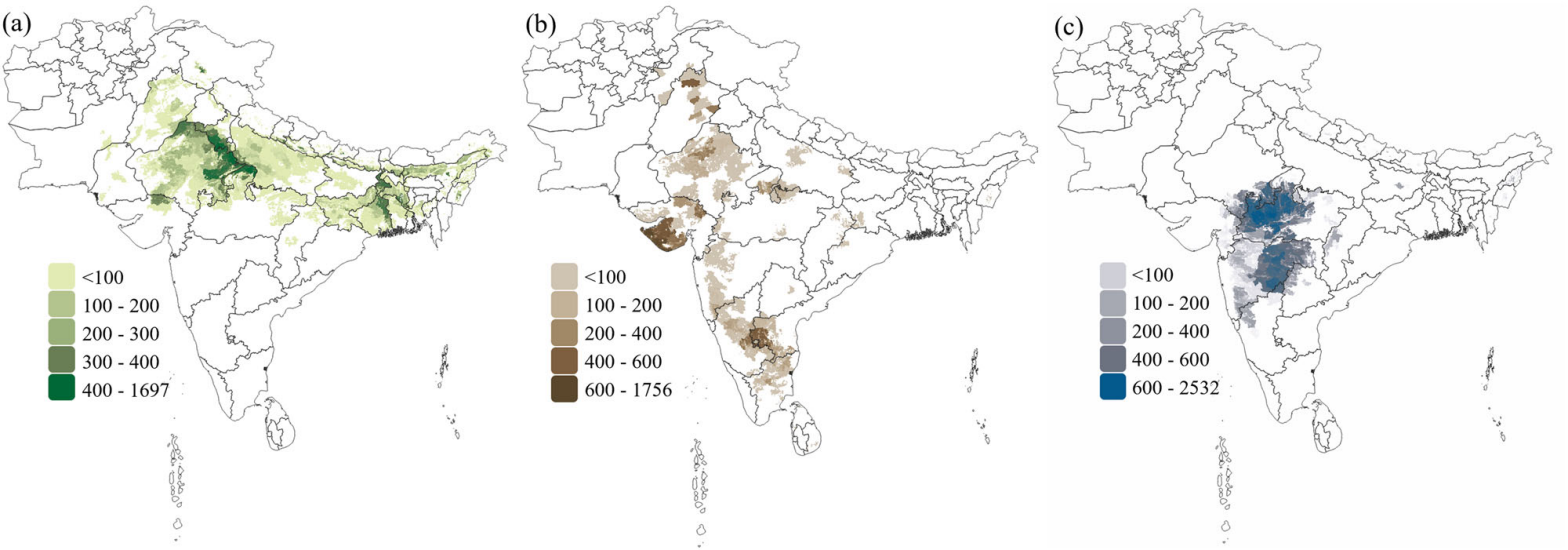
Spatial distribution of major oilseed crop areas across South Asia. Cropped area in hectares at a 0.05-degree resolution of major oilseed crops - (**a**) Mustard, (**b**) Groundnut, and (**c**) Soybean - in South Asia.

**Fig. 7:**
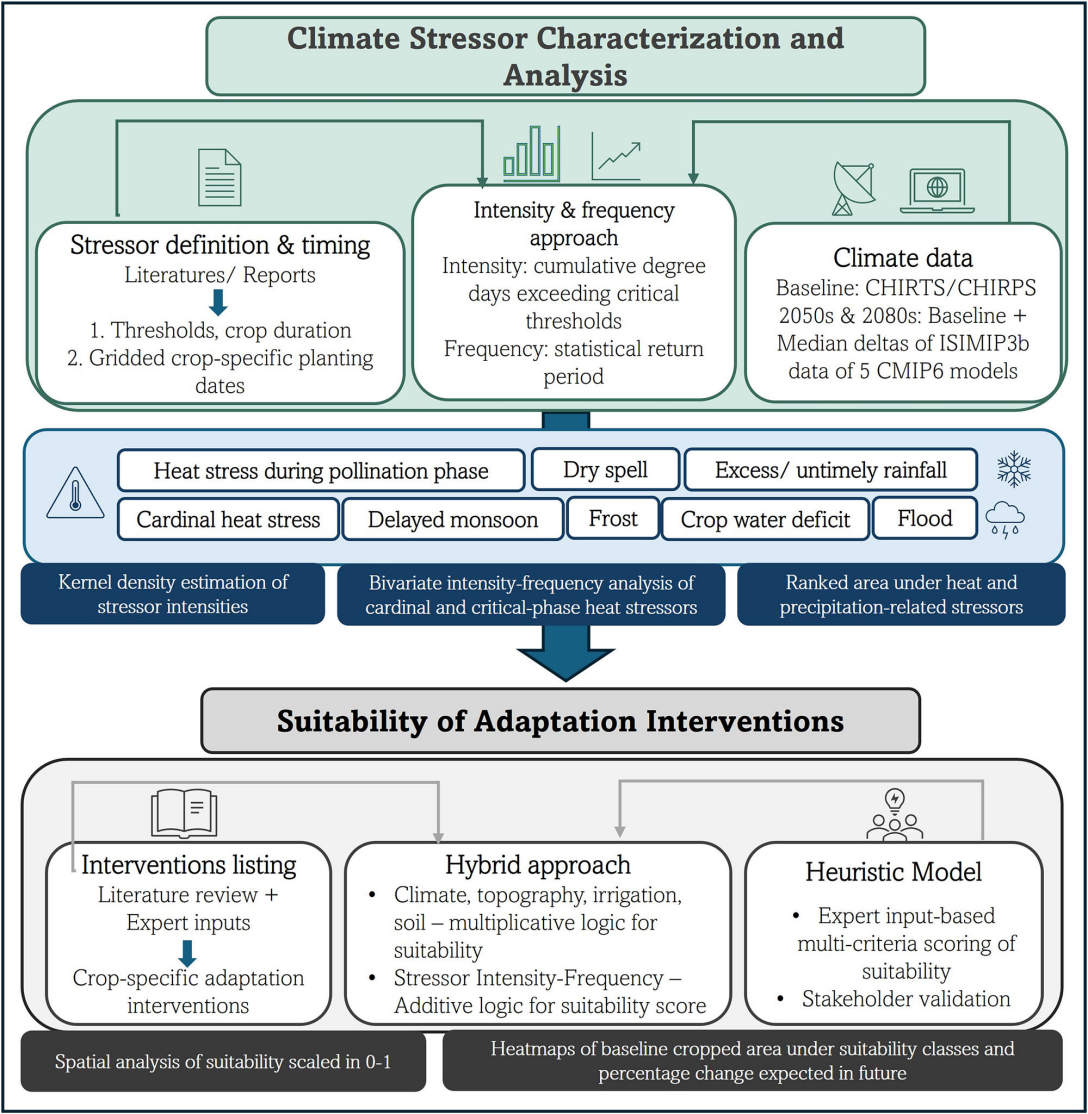
Conceptual framework linking climate stressor analysis to adaptation suitability assessment. The schematic diagram illustrates the overall methodology used to integrate climate stressor analysis with adaptation intervention assessment. Arrows represent the flow of data and processes, while boxes represent major analytical components.

In groundnut systems, in the high suitability category, genetic interventions showed the largest increase, with over 60% increase in area by the 2080 s. Crop insurance follows, with approximately a 30% increase (Fig. 7b). However, the direction and magnitude of change vary between SSP2–4.5 and SSP5–8.5 scenarios, indicating strong scenario dependence. For fertilizer intensification, a modest decline in the high-suitability area is observed, offset by an increase in the medium- and low-suitability classes. This suggests that the effectiveness of fertilizer-related interventions is expected to decline under future stress conditions (also similar to Figure 6ii). In the medium suitability category, broad-bed and furrow, fertilizer intensification, and precision fertilizer management show considerable increases in area share at the expense of low- and high-suitability classes. This pattern suggests a shift toward moderate suitability across much of the groundnut-growing regions. Micro-irrigation and precision water management show nonlinear trajectories. Under SSP5–8.5 in the 2050 s, both interventions gain area in the medium suitability class, but this reverses by the 2080 s as suitability decreases. This nonlinear behavior suggests that micro- and drip-irrigation systems become more effective under medium stress in the 2050 s, but effectiveness decreases as stressor intensity-frequencies exceed adaptive limits by the 2080 s. Overall, the results suggest that while several interventions, such as stress-tolerant varieties and crop insurance, retain potential under warmer climates, others, such as fertilizer intensification and irrigation interventions, become less suitable.

For soybean, crop insurance shows maximum change in area under the high suitability category, with more than a 60% increase by the 2080 s (Fig. 5c). This trend is consistent across both emission scenarios and exhibits very low scenario uncertainty. Under medium suitability, a positive response is observed in stress-tolerant varieties, broad-bed and furrow, and mulching under SSP5–8.5, while this trajectory is much weaker under SSP2–4.5, suggesting high scenario dependency. These interventions, therefore, indicate that structural and genetic measures will play an increasingly important role in maintaining soybean productivity under intensified stress conditions.

## Discussion

Changing climate stressors influence the effectiveness of current and future relevance of adaptation interventions in oilseed cropping systems across South Asia. We assessed multiple stressors, including heat and water deficits, frost, and rainfall extremes, for mustard, groundnut, and soybean under both baseline and projected scenarios. The analysis highlights changes in the intensity and frequency of stressors expected in future and the critical differences between full-cycle (cardinal) heat stress and the short-term stress experienced during the reproduction phase. The focus was to evaluate the climate-informed applicability of adaptation interventions such as genetic, agronomic, structural, and financial. We aimed to identify the interventions most likely to maintain or improve their effectiveness as the climate shifts in the future.

An increase in temperature in the future and modified rainfall regimes will increase heat and drought stress across the region^29,30^. The rightward shift in intensity distributions for both heat and the crop water deficit index across all three crops indicates expected increases in the intensity of heat and water deficit stressors in the future scenarios. Projected future increases in the intensity of heat-related stressors will affect the physiological characteristics of the oilseed crops. For mustard, heat and moisture stress are particularly critical during the flowering and early seed-filling stages. Even moderate warming during these phases reduces pollen viability and shortens grain filling, amplifying yield losses as warming accelerates across its major growing regions^5^. Exposure to low-temperature extremes during flowering and pod filling can still induce severe freeze injury in mustard, thereby leading to yield reductions^31^. Frost risk in mustard is expected to remain unchanged despite warming, indicating that mustard-growing regions in the north-western hilly parts of South Asia will continue to be frost-prone even in the future.

For soybean and groundnut, increasing CWDI intensity and lengthening dry spells indicate declining soil-moisture availability. This affects branching, pod initiation, and seed filling when water deficits disrupt carbon assimilation, nutrient uptake, and reproductive development^32,33^. In groundnut, temperatures above 35 °C reduce individual leaf area, decrease peg and pod numbers, and lower pod yield^34^. Temperatures beyond 34 °C induce pollen mortality^35^ and suppress leaf area and pod growth^36^. Elevated heat during pollination further reduces fertilization, increases kernel abortion, and ultimately lowers seed number and grain yield. Soybean can tolerate some heat, with thresholds around 34 °C during flowering, beyond which pollination success declines owing to changes in physiological and biochemical processes^6,37^. Heat stress during other stages reduces physiological activity and yield^38^. Interestingly, these C3 legumes, such as soybean and groundnut, exhibit relatively greater heat tolerance than several C4 crops^39^. However, rising heat-stress intensity in the future, especially during reproductive stages, will cause a significant decline in yield for these crops.

Excess rainfall and waterlogging are major constraints in soybean-growing areas of South Asia. Waterlogging severely affects soybean root morphology, growth, and yield, with losses of up to 80–90% when it occurs during sensitive mid-reproductive stages^9^. Despite future projections showing an overall increase in extreme events across regions, our study finds that in soybean-growing regions, extreme rainfall and waterlogging stress will likely decrease in the future. Although biotic stressors were not analysed in this study, emerging evidence shows that climate change is likely to intensify soybean vulnerability to major pests and diseases in South Asia. Rising temperatures, longer humid monsoon periods, and more frequent heat-humidity extremes are projected to increase infection pressure from fungal pathogens such as *Phakopsora pachyrhizi* (soybean rust), whose epidemic potential increases under warmer, wetter conditions^40^. These findings indicate that future soybean production will experience interacting abiotic and biotic pressures, increasing the need for advanced breeding and management strategies that address both stress domains^41^.

Our results also show major response differences between the full-cycle and the pollination-phase heat stress. Heat stress during the sensitive reproductive phase will be increasingly dominated by frequency-driven hazards rather than large shifts in intensity. In mustard, cardinal heat stress starts at around 22 °C, well below the 28 °C threshold for the pollination phase. However, in future, the area under pollination-phase heat stress is likely to double, caused by higher frequencies of the stressor. Legume plants such as soybean and groundnut are known to be relatively more tolerant to heat stress at the vegetative stage than at the reproductive stage^33^. This is also observed in our study. Moreover, we found that the changes in the intensities and frequencies of the stressors will be spatially distinct across South Asia in the future. In groundnut, the northern-cropped regions are exposed to intense cardinal as well as critical-phase heat stresses. On the other hand, the southern regions show frequency-driven stress patterns that imply reproductive-stage pressure even without extreme temperatures. Areas that currently face high-intensity heat stress will see even more intense heat in the future, while regions with frequent heat events will experience them more often. Increases in the frequency of heat events will induce more critical-phase heat stress compared with cardinal heat stress. In soybean, the pattern of intensities and frequencies of these stressors is noteworthy. By the 2050 s, both the frequency and intensity of cardinal heat stress are likely to increase. However, by the 2080 s, this stress becomes almost entirely frequency driven. Even though pollination-phase heat stress is more intense in the baseline than cardinal heat stress, we observe minor changes in the frequency of this stressor in the future.

Across all three oilseed crops, high-temperature–induced pollen sterility is projected to affect substantially larger areas, and this will emerge one of the important climate stressors. The high likelihood of repeated moderate critical phase heat events, rather than rare extremes, will increasingly impact the reproductive success of oilseed crops in the future. Hence, breeding efforts, agronomic scheduling, and climate-resilience interventions will need to explicitly address the critical phase heat hazard, as the existing adaptation measures do not adequately address it.

Mustard’s future suitability profile shifts toward nutrient-based and financial interventions, while structural measures and several irrigation options become less effective as climate stress intensifies. Fertilizer intensification and precision fertilizer management will sustain high suitability in those mustard-growing areas that are moderately stressed in the future. In these regions, improving nutrient use and N-response will positively impact yields^42,43^. Precision land levelling and broad-bed and furrow are useful in the Indo-Gangetic plains by improving water distribution and crop uniformity, but their benefits decline as heat and drought intensify beyond manageable limits. Mulching and targeted supplemental watering provide incremental buffering in moderate stress zones, but will not address reproductive losses when pollination-phase heat stress becomes more frequent in the future.

Groundnut and soybean cropping systems will primarily benefit from genetics and risk transfer-based interventions. Stress-tolerant varieties gain a large, high, and medium suitability area because regions under these crops are expected to shift from low to moderate stress in the future. This is supported by ref. ^44^, highlighting the substantial contribution of genetic strategies to climate resilience in these systems. However, varieties will need to be bred beyond their current tolerance limits to effectively counter projected extreme heat and water stress in some places. In groundnut, fertilizer intensification loses high-suitability area in the future as nutrient uptake decreases under severe moisture stress^45^. Irrigation and structural interventions are highly suitable in the baseline in groundnut and soybean systems. In future, these interventions show increases in the medium-suitability class when they can moderate soil moisture and microclimate effects of warming. However, their effectiveness decreases under very high water scarcity or extreme heat. It is important to note that our assessment of irrigation-based interventions assumes the current spatial pattern of irrigated versus rainfed production systems and does not explicitly account for future groundwater depletion or emerging competition for water resources. This static assumption is a limitation, as several regions of South Asia are projected to face severe declines in groundwater availability, which may constrain the scalability of irrigation-dependent adaptations. Incorporating future water-balance projections and basin-level water constraints will therefore be essential for refining the long-term suitability of irrigation-linked strategies^45^.

Crop insurance emerges in our analysis as a consistently high-suitability intervention with low scenario dependence, indicating its importance as climate stressors intensify in all three oilseed crops. This aligns with broader evidence that insurance functions not only as a risk-transfer instrument but also as a potent adaptation mechanism that helps farmers manage climate-induced yield variability^46^. It is important to note that, even though our analysis shows that crop insurance becomes more important under future warming, the real-world performance of insurance depends on far more than climate signals alone. Insurance must be able to translate climate stress into timely and accurate payouts. Poorly designed indices, or indices that fail to track shifting climate variability, can undermine their protective value by weakening farmer uptake^47^. Climate change will increase the need for insurance, but institutional design, index accuracy, and implementation efficiency will determine its actual adaptation value.

A major understanding from this analysis is the need to diversify and match adaptation portfolios with respect to both crop and location for various time slices and climate change scenarios in the future. Under moderate stressors in the future, most interventions retain value. When stressors become extreme under a high warming scenario, most interventions lose effectiveness. In such scenarios, relying solely on a single type of adaptation will be inadequate, especially in regions exposed to extreme stress. Multi-level policy coordination is needed to make adaptation options accessible and effective at the field level. It is important to note that the present analysis focuses on the climate stressor dimension, and does not explicitly simulate yield outcomes, which require integration of management, soil, and irrigation processes within crop modelling frameworks. Such assessments are being addressed as part of our broader work under the Atlas for Climate Adaptation in South Asian Agriculture (ACASA^48^). The framework presented here is intended as a screening tool to highlight where such coordination may be most needed. The trajectory of oilseed resilience depends on continued advances in genetics, the adoption of precision and sustainable management practices, investment in adaptive infrastructure, and the scaling up of risk-transfer tools.

Summarizing, this study focuses on three major oilseed cropping systems in South Asia, namely mustard, groundnut, and soybean. Existing assessments typically aggregate hazards, overlook crop-specific physiological thresholds, or provide broad estimates without linking them to practicable interventions. This work advances current understanding of climate-oilseeds interactions by moving beyond traditional impact assessments toward an adaptation-oriented, physiology-based framework. This framework is designed as a first-order screening approach to inform where different types of interventions may be more or less feasible under future climates. The novelty of this study lies in quantifying biologically relevant climate stressors, analysing intensity–frequency metrics for current and CMIP6-based futures, and linking the stressors to adaptation interventions using a logical, expert-reviewed heuristic framework.

Our results reveal several important patterns. First, the intensity of all heat-related stressors and the crop water deficit index is projected to increase in the future. Rainfall-related stressors, in contrast, show mixed responses owing to projected changes in future precipitation in the region. Second, heat stress during the full crop (cardinal) cycle and the pollination phase (critical phase) induces different responses under climate change. In particular, critical-phase heat stress is expected to increase primarily in frequency, raising the risk of more frequent stress conditions. In contrast, cardinal (full cycle) heat stress is projected to intensify more across oilseed regions. Third, genetic interventions and financial tools are likely to become increasingly important in the future across all three oilseed systems. Structural, nutrient, and irrigation-based interventions will likely become less effective under future climates as heat and moisture stresses exceed the thresholds these measures can realistically buffer. Also, their performance depends on the emission pathways. While our assessment focuses on the feasibility of different adaptation options, it is important to recognize that mitigation efforts to move onto lower-emission pathways remain the most effective means of reducing future climate risk. Without such action, the economic burden on farmers and governments, as well as the environmental costs of increasingly intensive adaptation, will be enormous.

Effective climate resilience for oilseeds in South Asia will require a flexible, dynamic portfolio of adaptation interventions that includes targeted genetic improvement, management practices (such as fertilizer and structural practices), and access to financial risk-transfer tools. The consistent finding that the effectiveness of most interventions declines under very high stress underscores the need for adaptive, location-specific strategies that can respond to the scale, timing, and frequency of future climate stresses. Also, our finding that future yield losses are likely to be governed by short, sensitive phenological windows rather than cumulative seasonal heat exposure leaves a major vulnerability unaddressed. As no existing adaptation intervention directly addresses these critical-phase heat stresses, we need to evaluate and develop interventions that can specifically protect pollination-stage processes under future climate change.

Even though this study offers a detailed assessment of climate stressors and adaptation suitability for major oilseeds, several limitations remain. First, while this analysis focuses on the major oilseed crops, several other oilseed species are grown in local, fragmented, and often marginal systems that remain unassessed. Second, while climate scenarios are spatially detailed, there is scope to use even higher-resolution projections to capture sub-regional variations. This approach is especially important for fragmented and diverse cropping systems. Also, adding more climate models to the analysis could provide a wider range of future possibilities and reduce uncertainty in the future scenario results. Third, the study does not explicitly account for socio-economic factors, barriers to adoption, or the scalability and economic viability of different interventions under farm-level realities. Future work should incorporate fragmented, minor oilseed crops, use next-generation downscaled scenarios for finer assessment, and layer in socio-economic dimensions to more closely align with IPCC risk framing and to inform policy and farm-level decision-making.

Despite these limitations, this work is an important step toward understanding the pathways of climate-induced variability in South Asian oilseed systems. The results are intended to guide prioritization which would help pave the way for prescriptive policy recommendations. By identifying the expected effectiveness of adaptation interventions under climate change scenarios, this study lays the groundwork for building robust, climate-smart oilseed production systems and highlights priority areas for future research.

## Methods

### Study area

South Asia is a globally important agricultural region supporting hundreds of millions of livelihoods. Favorable temperatures, fertile soils, and rainfall patterns enable high agricultural productivity and multiple cropping cycles. Mean annual temperatures range from about 20 °C in the northern plains to over 28 °C in the southern peninsular areas, making the region well-suited for diverse crop systems. These conditions make South Asia an agriculturally advanced and climate-sensitive region, ideal for examining how changing climate patterns may affect key crops.

Various edible oilseeds, such as mustard, groundnut, soybean, sunflower, sesame, safflower, castor, flaxseed, niger seed, coconut, and oil palm, are grown in South Asia. We focused on three major oilseed crops, mustard, groundnut, and soybean, that constitute about 33%, 17%, and 41% of South Asia’s total oilseed production, respectively, and are key to the oilseed economy (Fig. 6). About 6.95 million ha of mustard, 12.16 million ha of soybean, and 4.29 million ha of groundnut cultivation area are spread across the study area. Mustard is grown mainly in northern India during the *rabi* (winter) season, while groundnut and soybean are sown with the southwest monsoon in the *kharif* (summer) season. Using the MAPSPAM 2020^49^ and reported district-level crop area statistics, we calculated crop distributions at 0.05-degree resolution. For this, we first downscaled the MAPSPAM 2020 crop area data to 0.05-degree (~ 5 km grid) from its native 10 km (~0.1-degree) grid. The downscaling was performed in two steps. First, each 10-km MAPSPAM pixel was subdivided into the corresponding 5-km pixels, and the crop area was allocated to these smaller pixels in proportion to their geographic area within the original 10-km cell. This procedure preserves the relative spatial pattern of MAPSPAM at a finer resolution. Second, to ensure consistency with reported crop areas, we adjusted the aggregated 5 km crop areas within each district to match official district-level statistics wherever available. For pixels where district records were unavailable, we retained the MAPSPAM regridded data. This workflow maintains the spatial distribution from MAPSPAM while aligning district totals with official records.

These resultant distinct spatial and seasonal patterns in oilseed crops highlight how climate variability may affect each crop differently and guide necessary adaptation strategies.

### Climate forcing data

We used daily climate data on maximum temperature (Tmax), minimum temperature (Tmin), precipitation, and relative humidity. For the baseline period (1984–2013), we used datasets from the Climate Hazards Centre - CHIRTS (Climate Hazards Group InfraRed Temperature with Stations^50^) and CHIRPS (Climate Hazards Group InfraRed Precipitation with Stations^51^). We linear bias-corrected the CHIRTS Tmin using data from 600 meteorological stations to improve accuracy. For the 2050 s (2035–2064) and 2080 s (2065–2094), we used climate projections from five CMIP6 models: GFDL-ESM4, IPSL-CM6A-LR, MPI-ESM1-2-HR, MPI-ESM2-0, and UKESM1-0-LL. These coarse model data are bias-corrected and downscaled to 50 km by the ISIMIP (Inter-Sectoral Impact Model Intercomparison Project) group^52^. For the baseline, we computed the stressor intensities and frequencies using the CHIRTS and CHIRPS climate datasets for the 30-year period of 1984–2013. For the future, stressor intensities and frequencies were first computed for the SSP2-4.5 and SSP5-8.5 future scenarios for each ISIMIP model. We then computed deltas relative to the respective model baseline estimates and added these to the observed baseline climatology to produce future stressor maps. The selection of this delta approach ensures a spatially consistent, observation-driven baseline and reliable future climate information, suitable for assessing climate stressors and adaptation planning across South Asia.

### Climate stressors characterization and analysis

Our analytical framework directly links climate stressors with the assessment of technological interventions for oilseed crops (Fig. 7). We first identified key climate stressors based on physiological thresholds from the literature and expert inputs for each of the three oilseed crops. We quantified the climate stressors through intensities as well as frequency metrics to capture both magnitude and recurrence under baseline and future climate scenarios. The resulting stressor intensity-frequency classes formed the basis for evaluating adaptation interventions. Adaptation options were compiled from literature and expert consultation, and their suitability was assessed using a hybrid heuristic scoring approach. The calculation and analysis of climate stressors, as well as the analyses of adaptation interventions, are elaborated in the subsequent sections.

### Climate stressor analysis

We identified key climate stressors for mustard, groundnut, and soybean systems based on physiological thresholds and sensitive growth stages (Table 1 and Table 2). For each crop, we compiled experimental and physiological studies through a structured review of existing literature. We then identified biologically consistent thresholds by synthesizing reported values and using box-plot distributions to examine variability. For each stressor, the median value was selected as the representative threshold, which naturally reduces the influence of extreme values without requiring explicit removal of data points (Figs. 1S, 2S, and 3S). Details of these thresholds and literature sources are provided in Section 1 of the supplementary files. These consolidated thresholds form the basis for all intensity and frequency metrics. Intensity and frequency metrics were first computed and classified into five classes using k-means clustering. These were combined into a 5 × 5 matrix (Table 3), in which each intensity-frequency pair was assigned to a final severity class (Very Low, Low, Medium, High, Very High) according to a diagonal rule. Severity increases from the lower-left (low intensity, low frequency) to the upper-right (high intensity, high frequency).

**Table 1:** Definitions of heat-based climate stressors for mustard, groundnut, and soybean

Stressor	Definition	Mustard	Groundnut	Soybean
Heat stress	Intensity	Average cumulative stress degree days of Tmax > 22 ˚C for the entire crop period	Average cumulative stress degree days of Tmax > 35 ˚C for the entire crop period	Average cumulative stress degree days of Tmax > 36 ˚C for the entire crop period
	Frequency	Return period of Tmax > 22 ˚C events in 30-year period	Return period of Tmax > 35 ˚C events in 30-year period	Return period of Tmax > 36 ˚C events in 30-year period
High temperature-induced pollen sterility	Intensity	Average cumulative stress degree days of Tmax > 28˚C during the pollination period [30 -70 DAS].	Average cumulative stress degree days of Tmax > 34 ˚C during the pollination period [40–60 DAS].	Average cumulative stress degree days of Tmax > 34 ˚C during the pollination period [30-80 DAS].
	Frequency	Return period of such events in a 30-year period	Return period of such events in a 30-year period	Return period of such events in a 30-year period
Frost	Intensity	Average cumulative cold degree days of Tmin < 1 ˚C for the entire crop period	Not applicable	
	Frequency	Return period of such events in 30-year period		

Sources: see Section 1 of the supplementary file.

**Table 2:** Definition of precipitation-based climate stressors for mustard, groundnut, and soybean

Stressor	Definition	Mustard	Groundnut	Soybean
Delayed monsoon	Intensity	Not applicable	Average number of delay days counted from the mean onset^57^	
	Frequency		Return period of instances with delay > 7 days instances in 30-year period	
Dry spell	Intensity	Not applicable	Average dry spell score derived from^58^. The DS score is calculated as: $\mathrm{DS}\,{{\rm{Score}}}=\Sigma \exp \left(\frac{L}{14}-1\right)$ where *L* is the length (in days) of each dry spell of ≥ 14 days. Dry spells shorter than 14 days are not considered detrimental, while those longer than 42 days were truncated because stress beyond this duration is assumed to cause near-total yield loss. The DS score, therefore, increases non-linearly with dry-spell length, reflecting higher severity and agronomic stress associated with prolonged dry spells.	
	Frequency		Return period of instances with at least one 14-day long dry spell, corresponding to a DS score above 2.71	
Crop water deficit index (CWDI)	Intensity	Average seasonal Crop Water Deficit Index (CWDI; difference between seasonal water demand and supply) in mm. CWDI quantifies seasonal water deficit by comparing crop water demand against available water supply across spatially explicit grid cells where crop masks indicate crops’ presence. On demand side, we used spatially and temporarily varying evapotranspiration and fixed values for land preparation by crop. The reference evapotranspiration was estimated using elevation, daily temperature data and crop coefficients for each phenological stage using FAO-56 methodology^59^. On the supply side, we considered effective precipitation and soil-water holding capacity. For effective precipitation, we used USDA method^60^ and gridded daily precipitation data. We used soil water holding capacity based on crop rooting characteristics but assumed uniform soil properties within each depth zone. For rice, we used rooting depths limited to 30 cm and 120 cm for all other crops if the soil profile in that grid has depth of 120 cm else maximum depth of soil profile was considered. Besides, temperature, rainfall, elevation and soil-water holding capacity, the planting date parameter is also spatially varying. These calculations were made for all the grid cells where the crop mask indicates the presence of crops in that grid. Using crop masks and planting calendars, seasonal crop evapotranspiration (ETc), precipitation (PCPc), and effective precipitation were calculated and integrated with soil water holding capacity. CWDI is calculated using following formula CWDI = 1.2 × ETc + Land Preparation − Effective Precipitation − Soil Water Holding Capacity		
	Frequency	Return period of instances with deficit > 100 mm		
Excess rainfall	Intensity	Not applicable	Average of cumulative sum of rainfall above 100 mm for two consecutive days	
	Frequency		Return period of such instances in 30-year period	
Untimely rainfall	Intensity	Average of cumulative sum of rainfall above 100 mm for two consecutive days	Not applicable	
	Frequency	Return period of such instances in 30-year period		
Flood	Intensity	Not applicable	Average inundation days derived from the Global Flood Database (Dartmouth Flood Observatory^61^) for 2000–2018	
	Frequency		Return period of flood events

**Table 3:** Matrix for categorising stressor intensity and frequency into composite classes. VL, L, M, H, VH represents very low, low, medium, high and very high stressor intensity-frequency classes, respectively

		Intensity				
	Classes	1	2	3	4	5
Frequency	1	*VL*	*VL*	*VL*	*L*	*L*
	2	*VL*	*L*	*L*	*M*	*M*
	3	*VL*	*L*	*M*	*M*	*H*
	4	*L*	*M*	*M*	*H*	*VH*
	5	*L*	*M*	*H*	*VH*	*VH*

To capture complex stressor behavior, we normalized and compared their intensities across crops using ridge plots. These plots visualize how the kernel densities of normalized intensities of stressors behaved under baseline and the high-emissions future scenario (Shared Socio-economic Pathway^53^ (SSP) 5-8.5). We further analyzed joint variations in intensity and frequency of stressors through bivariate choropleth maps. Finally, ranked bump charts summarized the relative area affected under each stressor’s intensity-frequency class in the baseline and the future scenarios (SSP2-4.5 and SSP5-8.5). These analyses helped us understand the interplay between changes in the intensity and frequency of major climate stressors across the baseline and projected futures.

While CWDI integrates spatially explicit evapotranspiration, precipitation, effective rainfall, and soil water holding capacity, it is important to note that the CWDI estimates rely on simplified assumptions of ET₀ derived from temperature and elevation and may therefore not capture radiation or humidity-driven evaporative demand in all regions. The soil water holding capacity evaluation ignores heterogeneity in soil structure and organic matter content which may introduce local-scale uncertainty. This analysis focuses on relative changes of CWDI across scenarios rather than absolute values, these simplifications do not affect the direction of projected trends.

### Analysis of adaptation interventions

We developed a heuristic model to analyse the suitability of crop-specific adaptation options for mustard, groundnut, and soybean across South Asia.

The heuristic model follows a structured three-step framework. First, we identified adaptation interventions, focusing on documented practices that mitigate climate-related stressors for these crops (Table 4). Second, adaptation options were filtered based on a binary baseline agro-climatic suitability, including climate zone, soil characteristics, topography, and irrigation conditions (irrigated or rain-fed systems). For instance, irrigation-dependent interventions such as micro-irrigation, precision fertilizer management, and ICT-linked management were marked as suitable only in irrigated areas, while they were not applicable in rain-fed regions. This step helped us to identify where an intervention is technically feasible, forming the basis for subsequent stressor-specific scoring.

**Table 4:** List of adaptation interventions for mustard, groundnut, and soybean

Adaptation Intervention	Mustard	Groundnut	Soybean
Stress-tolerant varieties (STV)	√	√	√
Broad-bed and furrow (BBF)	√	√	√
Fertilizer intensification (FERTINT)	√	√	×
Supplemental Irrigation (SUPIR)	√	√	√
Crop Insurance (INSUR)	√	√	√
Precision Fertilizer Management (PFM)	√	√	×
Sprinkler Irrigation (SPRIR)	√	√	×
ICT-linked management (ICT)	√	√	×
Precision Water Management (DRPIR)	√	√	×
Precision land levelling (PLL)	√	×	×
Mulching (MULCH)	√	×	√

Third, a rule-based scoring system was applied to link stressor intensity-frequency classes with the effectiveness of each adaptation option. This step follows a heuristic approach, wherein generalized, literature-informed response functions are used to approximate how adaptation performance varies across stressor levels. In the absence of universally applicable empirical relationships, we developed simplified but biologically meaningful response patterns, represented through three distinct scoring rules:

- Bell-shaped suitability (for example, Stress-tolerant varieties, Supplemental irrigation): Maximum effectiveness under medium stress levels, with reduced applicability at very low or very high stress classes.
- Gradually decreasing suitability (for example, Broad-bed and furrow, Fertiliser intensification): Highest effectiveness under very low stress category, gradually declining as stress class increases, becoming negligible under very high stress.
- Uniform suitability (for example, Crop insurance): Applicable across low, medium, and high stressor classes, with zero suitability only in very low or very high stress classes.

Bell-shaped suitability was observed for interventions such as STV in groundnut, indicating that moderate climate stress levels are most effectively mitigated by stress-tolerant varieties. Minimal stress requires little intervention, whereas extreme stress surpasses the physiological buffering capacity of the variety^54^. Gradually decreasing suitability was applied to structural or agronomic practices such as DRPIR in mustard, where effectiveness is highest under very low stress conditions. Similarly, fertiliser intensification becomes less effective as the stresses increase^55^. The effectiveness declines progressively as stress increases due to limitations in water retention, soil management, or mechanistic constraints. Previous studies show that insurance influences farm resilience and is an adaptation strategy in agro-climatic contexts^56^. Hence, insurance is assigned a uniform suitability pattern across low–high stress classes. INSUR is least effective under negligible stress and overwhelmed under extreme stress, but provides consistent coverage across low-to-high stress scenarios.

Overall, in this framework, environmental suitability acts as a base filter, while stressor characteristics modify the effectiveness of interventions through predefined response functions. The resulting suitability scores therefore reflect both biophysical feasibility and climate-stressor-specific effectiveness.

Adaptation suitability at every crop growing pixel was computed using the suitability scores corresponding to stressor intensity-frequency (IF) classes. Each stressor was categorized into five classes on the basis of the K-means clustering of actual IF values. The heuristic model assigned technology-suitability scores (0-4) to each stressor IF class. For each pixel, the raw suitability score for each technology option ${a}$ was calculated as the sum of the scores across all stressors: ${R}_{a}={\sum }_{k=1}^{n}{s}_{k,a}$, where n is the number of stressors and s is the technology-suitability score. The raw scores were then normalized to a 0-1 scale using the maximum possible score for that option, ${S}_{a}=\frac{{R}_{a,\,\max }-{R}_{a}}{{R}_{a,\,\max }-{R}_{a,\min }}$, where ${R}_{a,\max }$ and ${R}_{a,\min }$ is the sum of the maximum and minimum attainable scores for each technology option, respectively. This scaling provides a comparable suitability index ranging from least (0) to most suitable (1). An illustrative example has been added for clarity.

Suppose for adaptation BBFIB (Broad-Bed Furrow), a pixel falls into the IF classes of stressors as in Table 5.

**Table 5:** Example of scoring a single pixel

Stressor	IF class	Score ${s}_{k,a}$(from heuristic table)
Frost (*n* = 1)	IF = 3	0
Heat stress (*n* = 2)	IF = 4	2
Untimely rainfall (*n* = 3)	IF = 2	2
CWDI (*n* = 4)	IF = 5	4
High temperature induced pollen sterility (*n* = 5)	IF = 1	0

Then, raw suitability:

$$R=0+2+2+4+0=8$$

Maximum possible raw score for BBFIB:

$${R}_{\max }=4+4+4+4+0=16$$

Scaled suitability:

$$S=\frac{16-8}{16-0}=0.5$$

So, this pixel has moderate suitability for BBFIB.

Using this method, suitability scores were calculated for every pixels and spatial maps were generated for the baseline and future SSP2-4.5 and SSP5-8.5 scenarios for the 2050 s and 2080 s. Heat maps summarized the proportion of crop area under low, medium, and high suitability in the baseline, and percentage changes in these classes under future scenarios, identifying which adaptation interventions are likely to become more or less effective.

## Supplementary information

Supplementary information

## Data Availability

The baseline climate data were obtained from the Climate Hazards Center repositories, including CHIRPS (https://www.chc.ucsb.edu/data/chirps3) and CHIRTS (https://www.chc.ucsb.edu/data/chirtsdaily). Future climate projections were sourced from the ISIMIP Phase 3b repository (https://data.isimip.org/search/tree/ISIMIP3b/InputData/climate/). All derived layers of climate stressors and adaptation interventions generated in this study are freely available through the Atlas of Climate Adaptation in South Asian Agriculture (ACASA) platform (https://acasa-bisa.org/).

## Code availability

The codes used for analysis in this study can be made available from the authors upon reasonable request.
