# Supplementary material for: Climate stressor projections inform adaptation needs in South Asian oilseed systems
Source: NPJ Sustain Agric. 2026 Jun 30;4(1):57. doi: 10.1038/s44264-026-00170-9 (PMC13318630; doi:10.1038/s44264-026-00170-9)
Supplement: Supplementary file 1 — Supplementary information [file 44264_2026_170_MOESM1_ESM.docx]

**Supplementary** **information**

*Climate stressor projections inform adaptation needs in South Asian oilseed systems*

Anasuya Barik ^[1]^, Paresh B Shirsath ^[1]^*, Roshan Babu Ojha ^[2]^, Vinay Kumar Sehgal ^[3]^, Virender Singh Bhatia ^[4]^, Sanjoy K Bandyopadhyay ^[1]^, Pramod K Aggarwal ^[1]^

*^[1]^ Borlaug Institute for South Asia (BISA), International Maize and Wheat Improvement Centre (CIMMYT), New Delhi, India*

*^[2]^ National Agricultural Environment Research Centre, Nepal Agricultural Research Council, Lalitpur, Nepal*

*^[3]^ Division of Agricultural Physics, ICAR-Indian Agricultural Research Institute, New Delhi, India*

*^[4]^ Division of Plant Physiology, Indian Institute of Soybean Research, Indore, Madhya Pradesh, India*

**Correspondence to P.Bhaskar@cgiar.org*

**Section 1. A**

**Temperature threshold for heat stress in Groundnut**

For the groundnut crop, the optimum temperature threshold value (median value) of 35°C shows the most favorable temperature during crop growth stages, with a lower quartile of 33.5°C and an upper quartile of 37°C, and the mean of the optimum temperature range is 35.2°C (Figure 1S (a)). Ketring (1984) reported that temperature >35°C decreases individual leaf area, reduces the number of pegs and pods, and results in lower pod yields of groundnut.


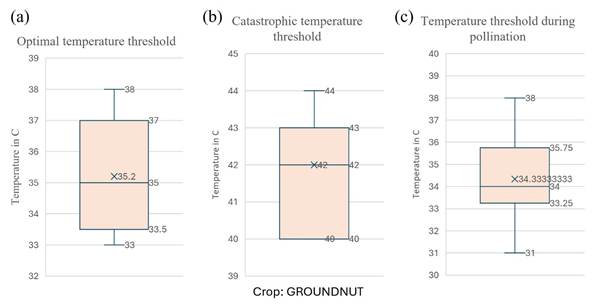


Figure 1S Box plots to derive thresholds of maximum temperature for (a) optimal growth during the entire crop cycle, (b) catastrophic effect during the entire crop growth period, and (c) catastrophic effect during the pollination period for groundnut

Studies on catastrophic heat stress show that in groundnut, maximum temperatures above 42°C during cardinal crop growth stages, with a lower quartile of 40°C and an upper quartile of 43°C, can lead to heat stress, resulting in significantly reduced physiological activities and grain yield (Figure 1S (b)). Studies also suggest that the maximum temperature beyond which pollination is affected in groundnut is 34°C (median value) as depicted in Figure 1S (c). The adverse effects may be associated with pollen mortality, which occurs at temperatures of 31-33°C. Cox (1979) reported that the critical maximum temperature during flowering is 34/30 °C, beyond which the leaf area and pod growth are reduced in the control environment. Higher heat stress during pollination period can reduce fertilization, cause kernel abortion and a reduction in the number of seeds and ultimately reduce grain yield in groundnut.

References (Groundnut):

1. Bright M.B.H.; Diedhiou I.; Bayala R.; Bogie N.; Chapuis-Lardy L.; Ghezzehei T.A.; Jourdan C.; Sambou D.M.; Ndour Y.B.; Cournac L.; Dick R.P. 2021 An overlooked local resource: Shrub-intercropping for food production, drought resistance and ecosystem restoration in the Sahel. Agriculture, Ecosystems and Environment, 319: 107523.

2. Cox, F. R. (1979). Effect of temperature treatment on peanut vegetative and fruit growth. Peanut science, 6(1), 14-17.

3. Kakani VG, Prasad PVV, Craufurd PQ, Wheeler TR (2002) Response of in vitro pollen germination and pollentube growth of groundnut (Arachis hypogaea L.) genotypes to temperature. Plant Cell and Environment 25: on flower production or the proportion of pegs forming pods, but did significantly reduce the proportion of flowers producing pegs. 25: 1651-1661.

4. Ketring, D. L. (1984). Temperature effects on vegetative and reproductive development of peanut 1, 2. Crop Science, 24(5), 877-882.

5. Mohamed. H.A. I984 Varietal differences in the temperature responses of germination and crop establishment. Ph.D. Thesis. University of Nottingham, UK.

6. Ong, C.K. (1986) Agroclimatological factors affecting phenology of groundnut. ICRISAT 1986, Agroclimatology of groundnut. Proceedings of an international symposium, 21-26 Aug 1985. ICISAT Shelian Centre, Niamev, Niger. ICRISAT, Patenceru, A.P. 502324, India

7. Prasad P.V.V., Craufurd P.Q., Summerfield R.J. & Wheeler T.R. (2000) Effects of short episodes of heat stress on flower production and fruit-set of groundnut (Arachis hypogaea L.). Journal of Experimental Botany 345, 777–784

8. Prasad PVV, Boote KJ, Allen LH Jr, Thomas JMG (2003) Supra-optimal temperatures are detrimental to peanut (Arachis hypogaea L) reproductive processes and yield at ambient and elevated carbon dioxide. Glob Change Biol 9:1775–1787

9. Prasad PVV, Craufurd PQ, Kakani VG, Wheeler TR, Boote KJ (2001) Influence of high temperature during pre- and post-anthesis stages of floral development on fruit-set and pollen germination in peanut. Aust J Plant Physiol 28:233–240

10. Prasad PVV, Craufurd PQ, Summerfield RJ (1999a) Sensitivity of peanut to timing of heat stress during reproductive development. Crop Science 39: 1352–1357

11. Prasad PVV, Craufurd PQ, Summerfield RJ, Wheeler TR(2000) Effects of short episodes of heat stress on flower production and fruit-set of groundnut (Arachis hypogaea L.). Journal of Experimental Botany 51: 777–784.

12. Vara Prasad P.V.; Craufurd P.Q.; Summerfield R.J. 1999 Fruit number in relation to pollen production and viability in groundnut exposed to short episodes of heat stress. Annals of Botany,84(3)381-386.

13. Wheeler T.R., Chatzialioglou A., Craufurd P.Q., Ellis R.H. & Summerfield R.J. (1997) Dry matter partitioning in groundnut exposed to high temperature stress. Crop Science 37, 1507– 1513).

**Section 1. B**

Temperature threshold for heat stress in Soybean

For soybean, the ideal growing conditions are below mean temperature of 35°C to ensure maximum productivity and minimize stress (Jumrani et al, 2022). The optimum temperature during the soybean crop growing period is shown in Figure 2S(a). While the statistical mean of the optimal temperature is 34.9 °C, we chose the median of 36°C to represent the threshold. This provides a better viewpoint of the central temperature without being skewed by extreme values. Studies indicate that, in soybeans, the catastrophic temperature threshold is 42 °C (with a lower quartile of 40 °C and an upper quartile of 43 °C; Figure 2S (b)) throughout the entire crop growth cycle. Beyond this temperature, heat stress causes a significant reduction in physiological activities and in soybean grain yield (Fig. 13).


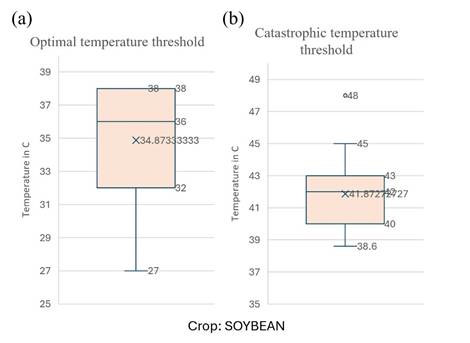


Figure 2S Box plots to derive thresholds of maximum temperature for (a) optimal growth during the entire crop cycle and (b) catastrophic effect during the entire crop growth period for soybean

The maximum temperature threshold for soybean during flowering datasets are very limited. However; typically beyond 34-35 °C, soybean plants experience significant reductions in pollination success due to heat stress, which impacts various physiological and biochemical processes (Gibson and Mullen, 1996; Jumrani and Bhatia, 2019).

References (Soybean):

1. Boote K.J. 2011 Improving Soybean Cultivars for Adaptation to Climate Change and Climate Variability. Crop Adaptation to Climate Change , ()370-395.

2. Cohen I.; Zandalinas S.I.; Fritschi F.B.; Sengupta S.; Fichman Y.; Azad R.K.; Mittler R. 2021 The impact of water deficit and heat stress combination on the molecular response, physiology, and seed production of soybean 172 1

3. DÂ‡vila-RamÂ’rez J.L.; AvendaÂ–o-Reyes L.; PeÂ–a-Ramos E.A.; Islava-Lagarda T.Y.; MacÂ’as-Cruz U.; Torrentera-Olivera N.G.; Rojo-Rubio R.; GonzÂ‡lez-RÂ’os H. 2018 Impact of zilpaterol hydrochloride and soybean-oil supplementation on intramuscular fat, fatty acid profile and cholesterol concentration in the longissimus muscle of male hair lamb under moderate heat-stress conditions 58 10

4. Djanaguiraman M.; Schapaugh W.; Fritschi F.; Nguyen H.; Prasad P.V.V. 2019 Reproductive success of soybean (Glycine max L. Merril) cultivars and exotic lines under high daytime temperature. Plant Cell and Environment , 42(1)321-336.

5. Ergo V.V.; Lascano R.; Vega C.R.C.; Parola R.; Carrera C.S. 2018 Heat and water stressed field-grown soybean: A multivariate study on the relationship between physiological-biochemical traits and yield 148

6. Ergo V.V.; Veas R.E.; Vega C.R.C.; Lascano R.; Carrera C.S. 2021 Leaf photosynthesis and senescence in heated and droughted field-grown soybean with contrasting seed protein concentration 166

7. Gibson L.R.; Mullen R.E. 2001 Mineral concentrations in soybean seed produced under high day and night temperature. Canadian Journal of Plant Science , 81(4)595-600.

8. Gibson, L. R., & Mullen, R. E. (1996). Influence of Day and Night Temperature on Soybean Seed Yield. Crop Science, 36(1), 98. doi:10.2135/cropsci1996.00112

9. Hamzaoui S.; Caja G.; Such X.; Albanell E.; Salama A.A.K. 2021 Effect of soybean oil supplementation on milk production, digestibility, and metabolism in dairy goats under thermoneutral and heat stress conditions 11 2

10. Heinemann A.B.; Maia A.D.H.N.; Dourado-Neto D.; Ingram K.T.; Hoogenboom G. 2006 Soybean (Glycine max (L.) Merr.) growth and development response to CO 2 enrichment under different temperature regimes. European Journal of Agronomy , 24(1)52-61.

11. Jumrani K.; Bhatia V.S. 2018 Combined effect of high temperature and water-deficit stress imposed at vegetative and reproductive stages on seed quality in soybean. Indian Journal of Plant Physiology , 23(2)227-244.

12. Jumrani, K.; Bhatia, V.S. 2019Combined effect of temperature and water stress on physiological and biochemical processes in soybean (Glycine max). Physiol Mol. Biol. Plants. 2019, 25, 697–712

13. Jumrani, K.; Bhatia, V.S. Combined effect of temperature and water stress on physiological and biochemical processes in soybean (Glycine max). Physiol Mol. Biol. Plants. 2019, 25, 697–711

14. Jumrani, K.; Bhatia, V.S.; Kataria, S.; Landi, M. 2022 Screening Soybean Genotypes for High-Temperature Tolerance by Maximin-Minimax Method Based on Yield Potential and Loss. Agronomy 2022, 12, 2854. https://doi.org/10.3390/agronomy12112855

15. Jumrani, K.; Bhatia, V.S.; Kataria, S.; Landi, M. Screening Soybean Genotypes for High-Temperature Tolerance by Maximin-Minimax Method Based on Yield Potential and Loss. Agronomy 2022, 12, 2854. https://doi.org/10.3390/agronomy12112854

16. Li J.; Wu M.; Chen H.; Liao W.; Yao S.; Wei Y.; Wang H.; Long Q.; Hu X.; Wang W.; Wang G.; Qiu L.; Wang X. 2024 An integrated physiological indicator and transcriptomic analysis reveals the response of soybean buds to high-temperature stress. BMC Plant Biology , 24(1)-.

17. Morrison M.J.; Cober E.R.; Saleem M.F.; McLaughlin N.B.; FrÂŽgeau-Reid J.; Ma B.L.; Woodrow L. 2010 Seasonal changes in temperature and precipitation influence isoflavone concentration in short-season soybean 117 1

18. Morrison M.J.; Cober E.R.; Saleem M.F.; McLaughlin N.B.; Frégeau-Reid J.; Ma B.L.; Woodrow L. 2010 Seasonal changes in temperature and precipitation influence isoflavone concentration in short-season soybean. Field Crops Research , 117(1)113-121.

19. Nakagawa A.C.S.; Ario N.; Tomita Y.; Tanaka S.; Murayama N.; Mizuta C.; Iwaya-Inoue M.; Ishibashi Y. 2020 High temperature during soybean seed development differentially alters lipid and protein metabolism 23 4

20. Narayanan S.; Zoong-Lwe Z.S.; Gandhi N.; Welti R.; Fallen B.; Smith J.R.; Rustgi S. 2020 Comparative lipidomic analysis reveals heat stress responses of two soybean genotypes differing in temperature sensitivity 9 4

21. Nehbandani A.; Filippi P.; Alizadeh-Dehkordi P.; Dadrasi A.; Soltani A. 2024 Use of interpretive machine learning and a crop model to investigate the impact of environment and management on soybean yield gap 75 1

22. Poudel S.; Adhikari B.; Dhillon J.; Reddy K.R.; Stetina S.R.; Bheemanahalli R. 2023 Quantifying the physiological, yield, and quality plasticity of Southern USA soybeans under heat stress 9

23. Ruchel Q.; ZandonÂ‡ R.R.; Fraga D.S.; Agostinetto D.; Langaro A.C. 2020 Effect of high temperature and recovery from stress on cropÃweed interaction 79 4

24. Schoving C.; Stöckle C.O.; Colombet C.; Champolivier L.; Debaeke P.; Maury P. 2020 Combining Simple Phenotyping and Photothermal Algorithm for the Prediction of Soybean Phenology: Application to a Range of Common Cultivars Grown in Europe. Frontiers in Plant Science , 10()-.

25. Schoving C.; Stöckle C.O.; Colombet C.; Champolivier L.; Debaeke P.; Maury P. 2020 Combining Simple Phenotyping and Photothermal Algorithm for the Prediction of Soybean Phenology: Application to a Range of Common Cultivars Grown in Europe. Frontiers in Plant Science , 10()-.

26. Schoving C.; Stöckle C.O.; Colombet C.; Champolivier L.; Debaeke P.; Maury P. 2020 Combining Simple Phenotyping and Photothermal Algorithm for the Prediction of Soybean Phenology: Application to a Range of Common Cultivars Grown in Europe. Frontiers in Plant Science , 10()-.

27. Schulte L.R.; Ballard T.; Samarakoon T.; Yao L.; Vadlani P.; Staggenborg S.; Rezac M. 2013 Increased growing temperature reduces content of polyunsaturated fatty acids in four oilseed crops. Industrial Crops and Products , 51()212-219.

28. Shaffique S.; Shah A.A.; Peter O.; Injamum-Ul-Hoque M.; Elansary H.O.; kang S.-M.; Al Azzawi T.N.I.; Yun B.-W.; Lee I.-J. 2024 The rhizobacterial Priestia megaterium strain SH-19 mitigates the hazardous effects of heat stress via an endogenous secondary metabolite elucidation network and molecular regulation signalling. BMC Plant Biology , 24(1)-.

29. Sicher R. 2013 Combined effects of CO2 enrichment and elevated growth temperatures on metabolites in soybean leaflets: Evidence for dynamic changes of TCA cycle intermediates 238 2

30. Tang R.; Seguin P.; Morrison M.J.; Fan S. 2021 Temperature and precipitation at specific growth stages influence soybean tocopherol and lutein concentrations 207 4

31. Vishwanath; Kumar S.; Purakayastha T.J.; Datta S.P.; Rosin K.G.; Mahapatra P.; Sinha S.K. 2021 Resistance and resilience of soil fluorescein diacetate activity against abiotic stress. Indian Journal of Agricultural Sciences , 91(8)1180-1184.)

**Section 1. C**

Temperature threshold for heat stress in Mustard

Literature suggests that occasional high temperature during sowing time and pod filling stage affects the productivity of the Mustard crop (Morrison & Stewart, 2002). According to the literature survey and the boxplot in Figure 3S(a), the optimal temperature range for these crops lies between approximately 20°C and 28°C, with a mean of 22.64°C. This range is crucial for vegetative growth, reproductive development, and ensuring efficient photosynthesis and biomass accumulation. Temperatures below this range can slow metabolic activity, whereas excessive heat may disrupt enzymatic processes.


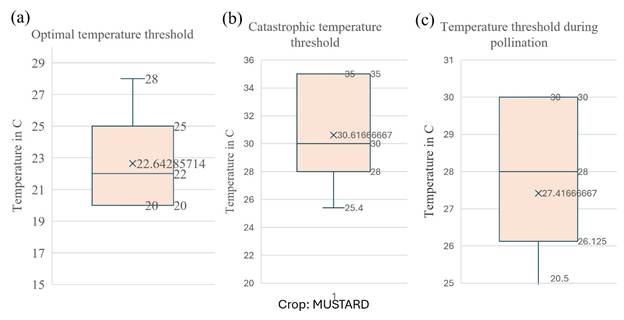


Figure 3S Box plots to derive thresholds of maximum temperature for (a) optimal growth during the entire crop cycle and (b) catastrophic effect during the entire crop growth period for mustard

The boxplots presentation indicate that the catastrophic temperature threshold for mustard is around 30°C (Figure 3S(b)). Scientists working on the thermal regime of 30-31°C of the mustard indicated that during the early vegetative stage, there is a significant positive correlation with the mustard yield (Rao et al, 2011 ). Exposure to temperatures beyond these limits can result in pollen sterility, reduced fertilization rates, and poor seed setting. Studies have shown that high temperatures above 30°C during reproductive stages accelerate senescence and shorten the grain-filling period, leading to lower yields (Angadi et al., 2000). Additionally, pollination is particularly sensitive to heat stress, with prolonged exposure above 27°C potentially causing flower abortion and reduced seed formation.

(Reference : Mustard and rapeseed)

1. Agarwal P.K.; Agarwal P.; Custers J.B.M.; Liu C.-M.; Bhojwani S.S. 2006 PCIB an antiauxin enhances microspore embryogenesis in microspore culture of Brassica juncea Plant Cell, Tissue and Organ Culture 86(2):201-210.

2. Anand A.; Nagarajan S.; Kishore N.; Verma A.P.S. 2010 Impact of high temperature at pod development stage on yield and quality of Brassica juncea cultivars under controlled conditions Indian Journal of Agricultural Sciences 80(12):1043-1047.

3. Angadi S.V.; Cutforth H.W.; Miller P.R.; McConkey B.G.; Entz M.H.; Brandt S.A.; Volkmar K.M. 2000 Response of three brassica species to high temperature stress during reproductive growth Canadian Journal of Plant Science 80(4):693-701.

4. Angadi S.V.; Cutforth H.W.; Miller P.R.; McConkey B.G.; Entz M.H.; Brandt S.A.; Volkmar K.M. 2000 Response of three brassica species to high temperature stress during reproductive growth Canadian Journal of Plant Science 80(4):693-701.

5. Chen, Sheng & Stefanova, Katia & Siddique, Kadambot & Cowling, Wallace. (2020). Transient daily heat stress during the early reproductive phase disrupts pod and seed development in Brassica napus L. Food and Energy Security. 10. e262. 10.1002/fes3.262.

6. Gan Y.; Angadi S.V.; Cutforth H.; Potts D.; Angadi V.V.; McDonald C.L. 2004 Canola and mustard response to short periods of temperature and water stress at different developmental stages Canadian Journal of Plant Science 84(3):697-704.

7. Hayat S.; Masood A.; Yusuf M.; Fariduddin Q.; Ahmad A. 2009 Growth of indian mustard (Brassica juncea L.) in response to salicylic acid under high-temperature stress Brazilian Journal of Plant Physiology 21(3):187-195.

8. Kacienė G.; Miškelytė D.; AbdElgawad H.; Beemster G.; Asard H.; Dikšaitytė A.; Žaltauskaitė J.; Sujetovienė G.; Januškaitienė I.; Juknys R. 2019 O3 pollution in a future climate increases the competition between summer rape and wild mustard Plant Physiology and Biochemistry 135():194-205.

9. Morrison, M. J., & Stewart, D. W. (2002). Heat stress during flowering in summer Brassica. Crop Science, 42(3), 797–803.

10. Patanè C.; Tringali S. 2011 Hydrotime Analysis of Ethiopian Mustard (Brassica carinata A. Braun) Seed Germination Under Different Temperatures Journal of Agronomy and Crop Science 197(2):94-102.

11. Rao, V. U. M., Rao, B. B., Nair, L., Singh, D., Sekhar, C., Venkateswarlu, B. 2011. Thermal sensitivity of mustard (Brassica juncea L.) crop in Haryana. J. Agrometeorol. 13(2): 131-134

12. (PDF) Impact of weather parameters on mustard yield in two diverse locations of India. Available from: https://www.researchgate.net/publication/369836737_Impact_of_weather_parameters_on_mustard_yield_in_two_diverse_locations_of_India [accessed Mar 03 2025].

13. Sehrawat A.; Sougrakpam Y.; Deswal R. 2019 Cold modulated nuclear S-nitrosoproteome analysis indicates redox modulation of novel Brassicaceae specific, myrosinase and napin in Brassica juncea Environmental and Experimental Botany 161():312-333.

14. Yaniv, Z., Schafferman, D., Zur, M., 1995. The effect of temperature on oil quality and yield parameter of high- and low-erucic acid Cruciferae seeds (rape andmustard). Ind. Crops Prod. 3, 247–252. )
